# Changes in Public Response Associated With Various COVID-19 Restrictions in Ontario, Canada: Observational Infoveillance Study Using Social Media Time Series Data

**DOI:** 10.2196/28716

**Published:** 2021-08-25

**Authors:** Antony Chum, Andrew Nielsen, Zachary Bellows, Eddie Farrell, Pierre-Nicolas Durette, Juan M Banda, Gerald Cupchik

**Affiliations:** 1 Department of Applied Health Sciences Brock University St. Catharines, ON Canada; 2 MAP Centre for Urban Health Solutions Unity Health Toronto Toronto, ON Canada; 3 Department of Psychology University of Toronto Toronto, ON Canada; 4 Department of Computer Science College of Arts and Sciences Georgia State University Atlanta, GA United States

**Keywords:** COVID-19, public opinion, social media, sentiment analysis, public health restrictions, infodemiology, infoveillance, coronavirus, evaluation

## Abstract

**Background:**

News media coverage of antimask protests, COVID-19 conspiracies, and pandemic politicization has overemphasized extreme views but has done little to represent views of the general public. Investigating the public’s response to various pandemic restrictions can provide a more balanced assessment of current views, allowing policy makers to craft better public health messages in anticipation of poor reactions to controversial restrictions.

**Objective:**

Using data from social media, this infoveillance study aims to understand the changes in public opinion associated with the implementation of COVID-19 restrictions (eg, business and school closures, regional lockdown differences, and additional public health restrictions, such as social distancing and masking).

**Methods:**

COVID-19–related tweets in Ontario (n=1,150,362) were collected based on keywords between March 12 and October 31, 2020. Sentiment scores were calculated using the VADER (Valence Aware Dictionary and Sentiment Reasoner) algorithm for each tweet to represent its negative to positive emotion. Public health restrictions were identified using government and news media websites. Dynamic regression models with autoregressive integrated moving average errors were used to examine the association between public health restrictions and changes in public opinion over time (ie, collective attention, aggregate positive sentiment, and level of disagreement), controlling for the effects of confounders (ie, daily COVID-19 case counts, holidays, and COVID-19–related official updates).

**Results:**

In addition to expected direct effects (eg, business closures led to decreased positive sentiment and increased disagreements), the impact of restrictions on public opinion was contextually driven. For example, the negative sentiment associated with business closures was reduced with higher COVID-19 case counts. While school closures and other restrictions (eg, masking, social distancing, and travel restrictions) generated increased collective attention, they did not have an effect on aggregate sentiment or the level of disagreement (ie, sentiment polarization). Partial (ie, region-targeted) lockdowns were associated with better public response (ie, higher number of tweets with net positive sentiment and lower levels of disagreement) compared to province-wide lockdowns.

**Conclusions:**

Our study demonstrates the feasibility of a rapid and flexible method of evaluating the public response to pandemic restrictions using near real-time social media data. This information can help public health practitioners and policy makers anticipate public response to future pandemic restrictions and ensure adequate resources are dedicated to addressing increases in negative sentiment and levels of disagreement in the face of scientifically informed, but controversial, restrictions.

## Introduction

### Background

Since the identification of SARS-CoV-2 in late 2019 until February 7, 2021, there have been 106 million cases of COVID-19 infections worldwide along with 2.32 million deaths. To contain the spread of infection, many national and regional governments have implemented a series of public health restrictions, including travel restrictions, closing of nonessential businesses, school closures, mandatory masking, social distancing rules, and other restrictions on the movement of populations.

While news media coverage of the public response to these COVID-19 restrictions have highlighted the growing number of antimask protests, COVID-19 conspiracies, and pandemic politicization with extreme views, these characterizations may not necessarily represent the general public opinion and sentiment about pandemic restrictions. The objective of this study is to investigate the association between pandemic restrictions and COVID-19–related public sentiment (ie, collective attention, aggregate sentiment, and sentiment polarization and disagreement) using Twitter data. The development of novel methods to incorporate sentiment analysis into the evaluation of public health restrictions is important, since traditional methods of monitoring public reactions are often expensive and inefficient (eg, random representative surveys) and may suffer from limited coverage and significant delays.

### Prior Relevant Studies

Previous research has emphasized that the use of social media by leaders and officials can lead to rapid dissemination of COVID-19–related information and influence of public policy [1]; however, the views of the general public, expressed via social media, should also be considered to inform effective pandemic response. Understanding how the public perceives these COVID-19 restrictions and information can inform public health messaging to maximize adherence to guidelines and reduce the spread of the virus. In a recent scoping review of studies related to COVID-19 and social media concerning the first outbreak from November 2019 to November 2020 [2], the authors noted a growing number of studies that document social media reaction to the COVID-19 pandemic to track and identify prevalent themes and concerns. While there is a larger body of literature that has identified changes in public opinions and perceptions over time using sentiment, topic, and content analysis of COVID-19–related social media content [3-8], the authors of the review noted that there is a scarcity of studies—at the time of publication in January 2021—that evaluate the impact of public health restrictions on public opinions (level of positive and negative emotions, level of disagreement, etc). However, some studies have begun to examine how COVID-19–related events (ie, COVID-19 case incidence, interventions, and news media) coincide with the frequency of COVID-19–related social media discussion (ie, collective attention).

In a study of COVID-19–related tweets from February 25 to March 30, 2020, in Belgium [9], researchers plotted tweet frequency alongside major COVID-19–related events, and they found that spikes in tweet frequency coincided with COVID-19 infections, stock market crashes, school closures, and infections of notable persons. The frequency of tweets about COVID-19–related topics has also been used to measure perceived susceptibility and severity of COVID-19 and was correlated with interventions, public events, and case counts [10]. Another descriptive study of COVID-19–related tweets from Australian states and territories detailed changes in aggregate sentiment trends in relation to COVID-19–related deaths and major COVID-19–related policy events [11] (eg, blocking arrivals from specific countries, expansion of testing criteria, and limits on outdoor gatherings). However, due to the lack of multivariate statistical modeling in the studies listed above, it was not possible to disentangle the independent contribution of these events on tweet frequency or aggregate sentiment and to investigate their relative importance in the shaping of public opinion.

Other studies have employed statistical models to understand factors that contribute to social media collective attention on COVID-19. In a study of the effects of COVID-19–related news coverage on collective attention [12]—measured by posts and comments on the r/coronavirus subreddit on reddit.com—between February 15 and May 15, 2020, researchers found, using linear regression, that the collective attention across the United Kingdom, the United States, Canada, and Italy was associated with daily COVID-19 incidence and COVID-19–related news articles. However, it is worth mentioning that the study did not include other factors that might also influence collective attention in their models, such as duration of business closure, the influence of holidays, and the introduction of restrictions including social distancing and mandatory face masks. The study focused mainly on collective attention (ie, comment and post frequency) but did not evaluate other indicators that might be more relevant to policy makers, such as the level of disagreement (eg, sentiment polarity) and aggregate sentiment (eg, positive to negative sentiment ratio) [4,5].

The limited number of studies that examined the association between COVID-19–related events, restrictions, and public opinion have typically approached the question from a descriptive manner, such as by graphically plotting major events and COVID-19 incidence on a timeline against COVID-19–related tweet frequency [9]. However, without considering the contribution of multiple factors simultaneously (business closures, school closures, holidays, other restrictions, etc), such as through the use of multivariate time series analysis, these studies may over- or understate the unique contribution of any given factor due to statistical confounding. To overcome this problem, our approach was informed by a prior study that used multivariate time series methods to analyze Twitter data, which accounted for multiple control variables, serial autocorrelation, and seasonal fluctuations and trends [13].

Additionally, the previous studies were unable to quantify the strength of the relationships between exposure (eg, days of business closure) and relevant public opinion outcomes (eg, level of negative sentiment). Our study will bridge this gap in the literature by using a dynamic regression approach to understand the unique contribution of restriction specifications (ie, business closures, school closures, announcements of masking and social distancing measures, and regional lockdown differences) on public opinion, while also taking into account the influence of contextual factors, including case counts, holidays, and COVID-19–related official updates. Our research question is as follows: What is the association between COVID-19 public health restrictions and measures of public opinion (ie, collective attention, positive to negative sentiment ratio, and level of disagreement) while accounting for potential confounding factors?

## Methods

### Twitter Data Collection

Data from our study were drawn from the largest COVID-19–related Twitter data set [14]. It was constructed using the following data-driven selection of keywords: COVD19, CoronavirusPandemic, COVID-19, 2019nCoV, CoronaOutbreak, coronavirus, WuhanVirus, covid19, coronaviruspandemic, covid-19, 2019ncov, and coronaoutbreak. The Social Media Mining Toolkit [15] was used to collect all tweets worldwide with the keywords mentioned above starting on March 12, 2020. Further details about the data collection process can be found in a previous paper [14]. We used the cleaned data set of English-only tweets, with retweets filtered out. A retweet is the sharing of a tweet without any added comments; however, quoted tweets (ie, sharing a previous tweet along with one’s own comment) were included in the data.

To identify a subset of tweets originating from Ontario, Canada, geographic coordinates were used for tweets with geolocation enabled. For tweets that did not have geolocation enabled, our team created an algorithm that matched the text of the user-defined location to a standard gazetteer at GeoNames [16]. The gazetteer data contain alternative spellings for cities across different languages and include various airport codes used for matching (eg, YTO and YYZ for Toronto). We used a list of locations that had a population of 1000 or greater. When inferring location based on user input, our algorithm matches to a city with a unique name. For cities that share the same name with other cities, the algorithm attempts to find a match based on country and/or state identifiers in the text. If there is no state or country data in the text (eg, “London” only), the tweet is matched to the place with the highest population; in this case it would be London, England, UK. Matching to the largest population center, in cases where no further information is available, was based on the assumption that people from the largest cities are more likely to leave out further country or regional identifiers, while those in smaller cities that share the same name with larger cities are more likely to include further regional information. If no match is made at the city or town level, the text is then matched to a higher-level geographical unit (ie, state, region, or province) and then to a country. Out of the subset of all tweets with user-entered location text, our program matched 89.9% to a GeoName ID. A link to our GitHub repository for the algorithm is available [17]. Our program also examined any Unicode data (eg, a flag emoji) entered by users in lieu of country-level information. We randomly sampled 250 matches to ensure that the matches were made according to the algorithm described above. In total, we identified 2,649,317 tweets originating from Canada between March 12 and October 31, 2020, 43.4% of which (1,150,362 tweets) were from Ontario.

### Sentiment Analysis

Once we collected the COVID-19 Twitter data, we conducted sentiment analysis using the VADER (Valence Aware Dictionary and Sentiment Reasoner) algorithm, which assigned a sentiment score (–1 to +1) to each tweet that represents a polarity—negative or positive—and a strength of emotion for the tweets. Table 1 presents examples of positive, neutral, and negative tweets and their VADER-assigned sentiment scores. In a prior study [18], scoring by the program had an *r*=0.88 correlation with gold-standard ground truth (ie, the mean sentiment rating from 20 prescreened and appropriately trained human raters). Scores of –0.05 and under were negative, scores of +0.05 and above were positive, and scores in between were neutral. These thresholds are conventional for studies using VADER [19,20], and classification by human raters was found to be well-matched to VADER results when using these scoring thresholds [21].

**Table 1:** Examples of positive, neutral, and negative tweets with VADER^a^-assigned sentiment scores.

Sentiment score	Classification	Tweet
0.93	Positive	“Thank you so much @johnkrasinski for this series! I think it helped remind everyone how much good there is in the world. I really hope the silver lining of COVID-19 is people continue to be kinder to one another and truly realize we're all in this together.”
0.65	Positive	“@celliottability notes that Ontario has made great strides on COVID-19 testing and contact tracing. Anyone who wants to get a COVID-19 test can do so, even if they don’t have symptoms”
0.03	Neutral	“#SSHRCResearchers Helen Kennedy and Sarah Atkinson look at how the industry is adapting to the new reality of #COVID19”
–0.04	Neutral	“Why you should wear a #mask #COVID10 @ottawahealth”
–0.40	Negative	“COVID-19 Compliance: One-in-five Canadians making little to no effort to stop coronavirus spread”
–0.57	Negative	“Because the Chinese just hate witchcraft. Riiiiight... Cough, feng shui, cough #WuhanVirus #COVID19”

^a^VADER: Valence Aware Dictionary and Sentiment Reasoner.

### Study Outcomes

#### Overview

To study public opinion on COVID-19–related public restrictions using Twitter data in a comprehensive manner, we considered (1) the collective attention on COVID-19 measured by the level of COVID-19–related discussion (ie, COVID-19–related tweet frequency); (2) the aggregated sentiment level, measured using a positive to negative sentiment ratio; and (3) the level of disagreement, or sentiment polarity, measured by the Gini index.

#### COVID-19–Related Discussion: Tweet Frequency

We used tweet frequency to represent the level of participation in COVID-19–related discussion on Twitter on a specific day. Prior studies have utilized social media activity data (ie, Twitter and Weibo post frequency) to identify collective attention with regard to COVID-19 interventions and events [5,12]. We have included tweet frequency to estimate how public health restriction can influence COVID-19–related collective attention, which may provide a useful metric that policy makers can use to identify potential areas of concern at the population level.

#### Aggregate Sentiment

To determine the aggregate sentiment of a particular day, a value was derived for each day that represents the ratio of positive to negative sentiment, expressed by the following:


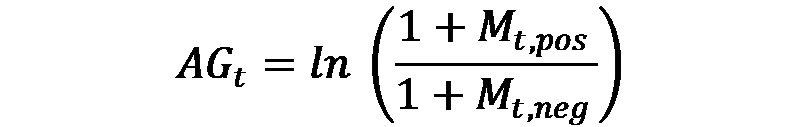


where M_t,pos_ is the total count of positive tweets with sentiment scores greater than 0.05, and M_t,neg_ is the count of negative tweets with sentiment scores lower than –0.05. The natural log transformation is used to avoid excessively large ratios. This specific formula for sentiment aggregation to measure the net positive sentiment has been commonly used in prior literature of Twitter sentiment analysis, since it takes into account the number of Twitter users on a given day [22,23]. The use of positive to negative sentiment ratios have been predictive of group-level behaviors, such as stock market [23] and movie-going behaviors [24,25]. These previous studies excluded neutral tweets, since they tend to represent objective or informational statements, often coming from nonprofits, governments, or academic institutions. We did not include them in our measure, since we aimed to measure the subjective perspectives and views of individuals characterized by negative and positive emotions. In addition, a public health restriction that is associated with significantly more positive than negative tweets has a greater chance of being accepted and may reflect a higher level of public compliance.

#### Gini Index

**Figure 1:**
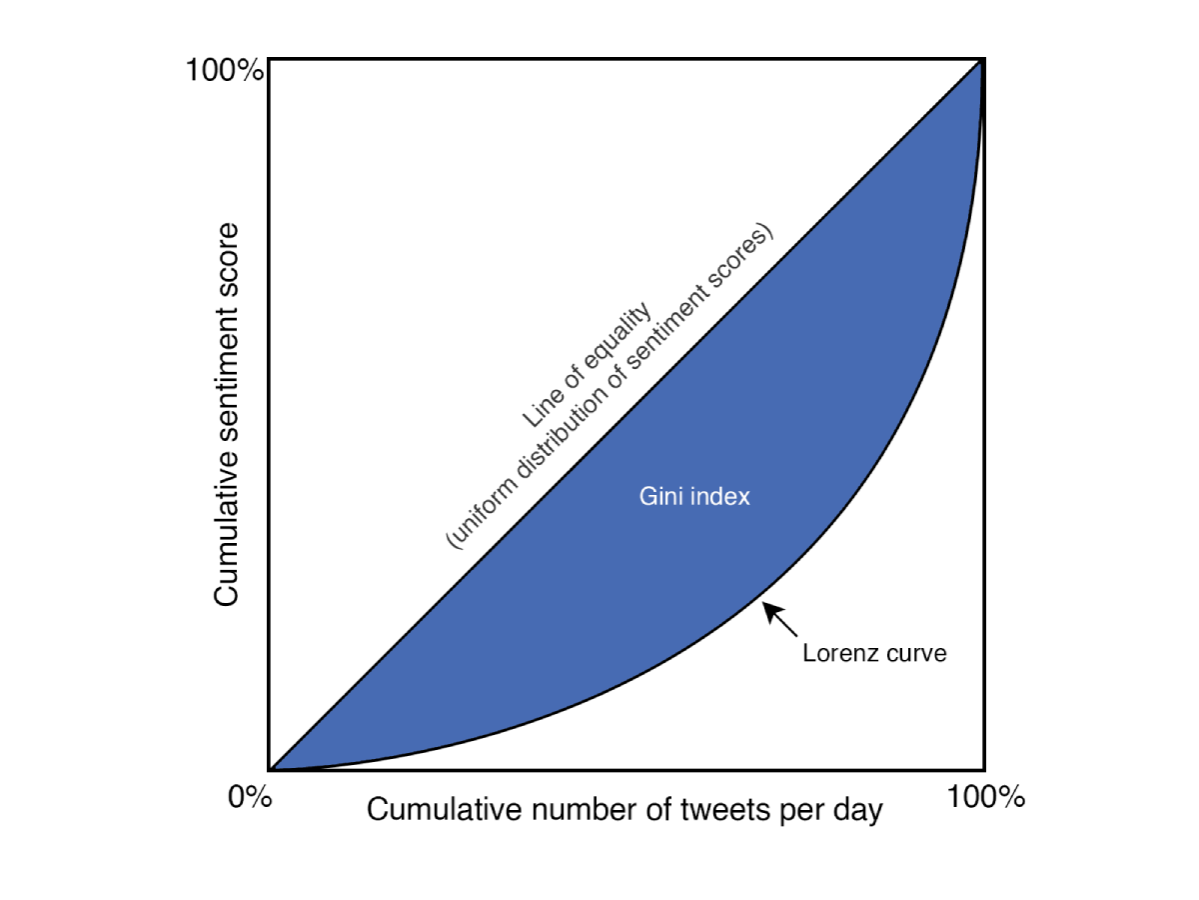
Graphical representation of using the Gini index to measure sentiment disparity.

A Gini index was derived to measure the level of disagreement, or sentiment polarization, in COVID-19–related tweets. Although the Gini index is typically used in the literature to describe income inequality, this index has been used to measure inequality in other areas of social interest, such as opportunity for social mobility [26], educational attainment [27], public transit availability [28], and movie preferences [29]. A Gini index of zero represents the lowest level of disagreement (ie, perfect equality of scores), and a higher Gini index represents greater differences in the sentiment scores across tweets on a particular day. For example, a Gini index of 0.30 means that 30% of the sentiment scores would have to be redistributed in order for everyone’s score to be the same. The Gini index is calculated based on the area between (1) the line of equality and (2) the Lorenz curve, as shown in Figure 1.

The 45° line of equality, where x=y, is the hypothetical situation of uniform distribution where each tweet, in the same day, exhibits the same sentiment score. By plotting the cumulative sentiment score on a given day against the cumulative number of tweets, the Lorenz curve can be used to characterize sentiment score disparity (ie, visually represent how a range of tweets, from those with the lowest to highest sentiment scores, contribute to the relative increases in the cumulative score, where a more concave Lorenz curve represents greater disparity). To create a daily Lorenz curve, we started by rescaling each tweet-level sentiment score (–1 to +1) to a range from 1 to 100, because the standard calculations cannot include negative values. We then ordered tweets from the lowest to the highest sentiment scores and plotted the cumulative number of tweets against the cumulative tweet sentiment score. Next, the Gini index was calculated by finding the area under the line of equality and above the Lorenz curve (eg, shaded blue area in Figure 1). This method of calculating a Lorenz curve and Gini index was repeated for each day in our data set. Our Gini index (G) is calculated using the following equation:


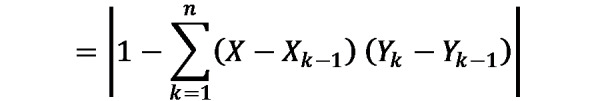


where X_k_ is the cumulative proportion of tweets over n number of tweets in a given day (from k=0,...n,), and Y_k_ is the cumulative proportion of sentiments in a given day.

### Creating the Ontario COVID-19 Timeline

#### Overview

We created a comprehensive timeline of COVID-19–related restrictions and events in Ontario by consulting with the COVID-19 intervention timeline created by the Canadian Institute for Health Information [30], the timeline of COVID-19 events created by Public Health Ontario [31], and timelines that were created by news media [32,33]. This full timeline used for the study is available in Multimedia Appendix 1. In Ontario, key events on the timeline include the following:

1. The declaration of a state of emergency on March 17, 2020, which led to the closing of all nonessential businesses and schools.
2. The closure of the US-Canada border to nonessential travelers on March 21, 2020.
3. The partial reopening of selected regions in Ontario that began on June 12, 2020.
4. The reopening of nearly all businesses and public places across Ontario, with restrictions, by August 12, 2020.
5. The restrictions to reduced private gatherings that were reinstated on September 19, 2020.
6. The restrictions on restaurants, bars, banquet halls, and gyms that were reinstated on October 3, 2020, in selected urban regions.

For the purpose of our study, we focused on four dimensions of the public health restrictions, including (1) business closures, (2) school closures, (3) regional lockdown differences (ie, partial vs province-wide lockdown), and (4) additional public health measures (eg, travel, social distancing, and masking).

#### Business Closures

Ontario implemented closures and limitations on nonessential businesses to help control the spread of COVID-19. With the exception of essential businesses—including stores that sold food, big box retailers, pharmacies, and alcohol stores—that stayed open, many businesses were closed or offered limited services (eg, restaurants were limited to providing delivery or take-out services only). Given the importance of business and retail services to Ontario residents, we considered the cumulative effect of business closures. An urban-centric approach was used to define business closure, since the majority of Ontarians live in major urban centers (71.7% as of 2019) [34]. While rural areas reopened earlier in the summer of 2020, for the purposes of the timeline, we did not consider businesses across the province to be reopened until it was the case in all major population centers, with some limits on capacities. To account for the influence of the earlier rural reopening, we included an adjustment variable to indicate the partial reopening of Ontario. To construct the business closure variable, we first created a binary variable to indicate, for each day on the timeline, whether nonessential businesses were closed due to restrictions. For each consecutive day of closure, we created a cumulative variable to consider effects associated with the duration of closure (eg, 1 for the first day of closure and 10 for the 10th day of closure). Additionally, we hypothesized that each additional day of closure had an additive but diminishing effect (ie, logarithmic growth) because each additional day of the closure could have a normalizing effect due to adaptation; therefore, we derived the natural log cumulative business closure variable to be used in our regression models.

#### School Closures

We considered primary and secondary school closures to be a significant restriction that impacts a large number of Ontario families. Moreover, the closure of primary and secondary schools would lead to the need for parents to make accommodations to provide childcare. Days for school closure due to COVID-19 restrictions were represented through a binary variable. Universities and colleges were not considered, as students are older and able to care for themselves, therefore causing less disruption. We hypothesized a logarithmic growth effect on the experience of school closure because each additional day of the closure could have a normalizing effect, where each additional day of closure has an additive but diminishing effect, due to adaptation and adjustment to new childcare arrangements and work accommodations.

#### Regional Lockdown Differences

Over the course of the study period, Ontario implemented province-wide lockdowns and partial lockdowns, where the latter focused on dense urban areas (eg, Toronto, Peel, and Ottawa) to implement a targeted approach to pandemic restrictions. We categorized days in our time series into three groups: (1) province-wide lockdown, (2) partial lockdown, and (3) no lockdown. We expected these variations in lockdown conditions to have an effect on social media discussion and sentiment. Decisions around the implementation of partial versus province-wide lockdowns were controversial [35], with diverging beliefs around the benefits of a province-wide lockdown (eg, under partial lockdown, some people may travel to an adjacent region with no lockdown to visit the gym) and the benefits of partial lockdowns (eg, partial lockdown allows for a flexible approach tailored to local COVID-19 infection rates to minimize economic impacts).

#### Additional Public Health Measures

Between March 12 and October 31, 2020, there were a number of additional restrictions put in place by the Ontario and federal governments to reduce the spread of COVID-19. These include measures such as nonessential travel restrictions (eg, US-Canada border), mandatory quarantine for travelers, limits on indoor and outdoor gatherings, health and safety bylaws for businesses such as sanitizer and plexiglass, and social distancing and mask policies for the general population. Given the overlapping nature of multiple public health measures, which often target specific concerns (ie, travel, distancing, and hygiene), we only considered the day a restriction was announced. Unlike business and school closures, these additional public health measures remained enforced for the duration of our data set. We characterized each day as having either (1) a new or updated restriction announced or (2) no restrictions announced.

#### Control Variables

In order to adjust for other contextual factors that may also influence COVID-19–related public opinion, we included the following variables as control factors: (1) COVID-19–related official updates, (2) statutory holidays, (3) COVID-19 daily incidence for Ontario, and (4) COVID-19 daily incidence for Canada, excluding Ontario.

#### COVID-19–Related Official Updates

Multiple official COVID-19–development announcements have been released over the course of the pandemic, including press conferences for major events (ie, case counts and mortality milestones), new screening guidelines, and provincial reopening plans, as well as notable COVID-19 developments (eg, new evidence on the effectiveness of nonmedical masks) from the World Health Organization, Ontario Hospital Administration, and government officials. While additional public health measures detail restrictions enforced on the population, COVID-19–related official updates are meant only to provide useful information about COVID-19 events. For example, after the announcement that Canada had surpassed 100,000 COVID-19 cases, we might expect that people would take to social media to express their emotions about this information.

#### Statutory Holidays

There is prior evidence that public sentiment and frequency of posts on holidays systematically differ from those on nonholidays [36]. There were also public concerns that travel and social gathering plans over holidays, and long weekends, may promote COVID-19 infections [37] and, in turn, public sentiment concerning COVID-19. Therefore, we included Canadian statutory holidays in our models as an adjustment variable. In our study period, seven holidays in 2020 were identified: Good Friday (April 10), Easter (April 12), Victoria Day (May 18), Canada Day (July 1), Civic Holiday (August 3), Labour Day (September 7), and Thanksgiving (October 12). If the holiday was part of a long weekend, the entire weekend was coded as a holiday. For example, Labour Day was on Monday, September 7; therefore, Saturday, September 5 and Sunday, September 6 were also coded as a holiday.

#### COVID-19 Daily Incidence

COVID-19 new daily case counts at the provincial and national levels were a major focus in news media and a significant factor that could influence public opinion and collective attention on COVID-19. Case information is based on the Public Health Case and Contact Management Solution [31], which is Ontario’s primary disease-reporting system. Case counts for Canada were drawn from the COVID-19 Data Repository at Johns Hopkins University [38]. We subtracted the Ontario case counts from the Canada case counts so the national numbers were deduplicated.

### Statistical Analysis

Our study combined an autoregressive integrated moving average (ARIMA) approach to time series modeling with regression methods in order to examine the associations between public health restrictions and changes in sentiment measures over time [39]. These are generally known as dynamic regression models, and they are typically used to generate forecasts [40] but are also useful for the purpose of explanatory modeling (ie, understanding the relationship between multiple time series variables), as is the purpose of our study. They take on the form where the outcome time series y_t_ is modeled as a function of k explanatory variables (x_1,t_,...x_k,t_), where n_t_ is allowed to be autocorrelated, using ARIMA errors:


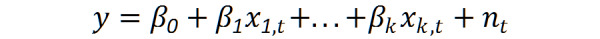


The ARIMA error n_t_ may contain (1) autoregressive (AR) terms used to determine the relationship between the current observation and previous observations, (2) moving average (MA) terms to determine the relationship between current observation and previous error, and (3) differencing terms to stationarize the time series outcome if necessary. For parameter estimations of ARIMA error terms (ie, the content of n_t_), the auto.arima() function was used in the *forecast* package in R, version 4.0.3 (The R Foundation). The purpose of using this function is to fit the most appropriate ARIMA model according to Akaike information criterion (AIC) values; the AIC quantifies the model’s goodness of fit (ie, lower is a better fit). The function searches across a number of candidate models, selects the appropriate number of AR and MA terms based on minimization of the AIC, and applies the appropriate number of differencing terms to stationarize the outcome time series [41]. The selection of terms is denoted using (*p, d, q*), where p is the number of AR terms, d is the degree of differencing, and q is the number of MA terms. For example, if auto.arima() determined that ARIMA (1, 0, 0) was most appropriate for the dynamic regression model, this would indicate that a model using one AR term produces the greatest minimization of the AIC.

We constructed three models, with Model 1 for the frequency of tweets concerning COVID-19 each day (ie, collective attention), Model 2 for the aggregate sentiment score representing the ratio of positive to negative tweets each day, and Model 3 for the level of sentiment disparity within each day, using the Gini index. The outcomes were deseasonalized using the ts() function in R, since there is a tendency for more Twitter activities on weekdays over weekends. Following the deseasonalizing procedure, we used the augmented Dickey-Fuller and Kwiatkowski-Phillips-Schmidt-Shin tests for stationarity. If the trend was not stationary, differencing for stationarity would be handled by the auto.arima() function. Each outcome was regressed on all seven predictors mentioned above, and regressors that were not significantly associated with the outcome (at *P*<.05) were subsequently removed. All statistical analyses were completed on RStudio Cloud (updated to January 20, 2021) using R, version 4.0.3.

## Results

### Overview

**Figure 2:**
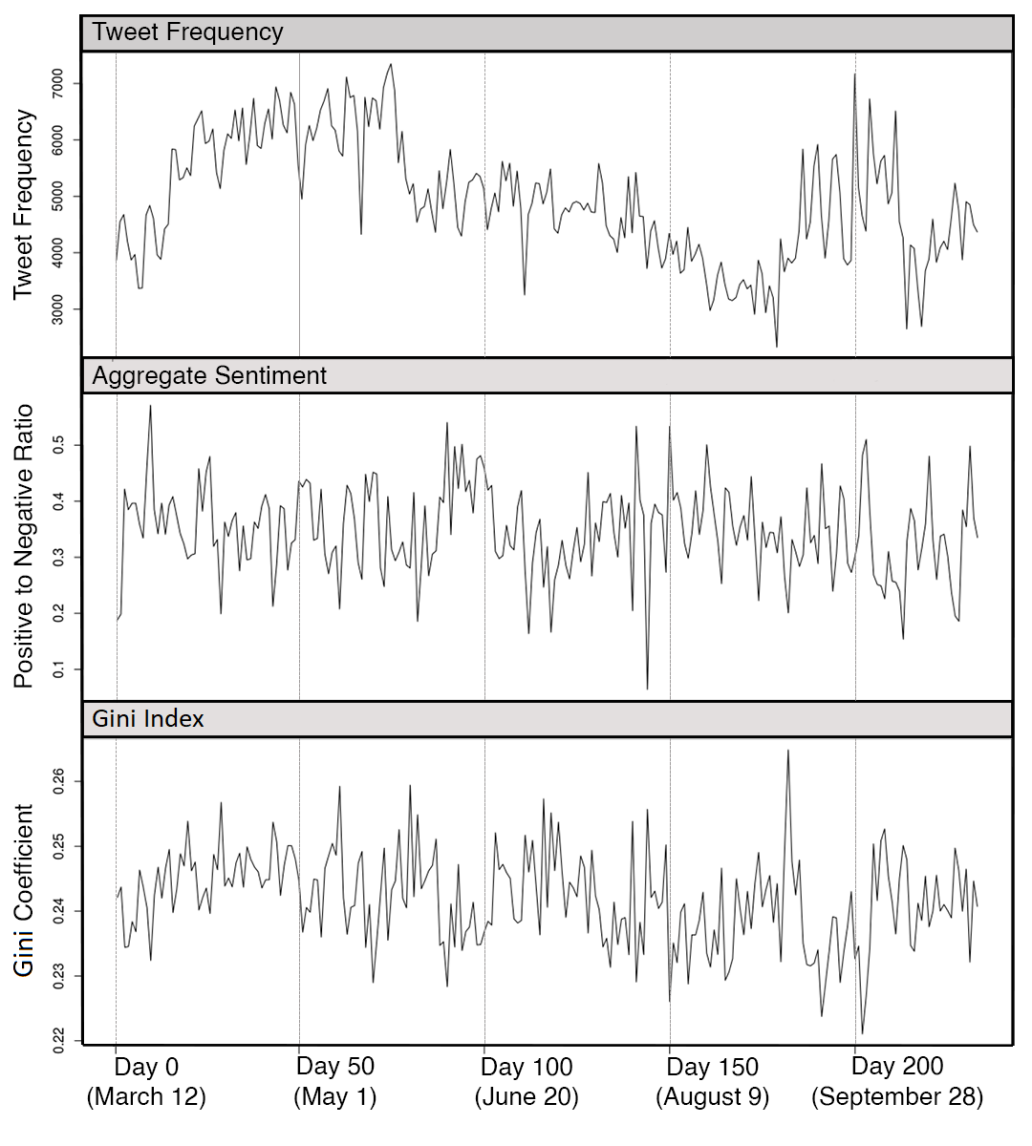
Daily tweet frequency, aggregate sentiment, and Gini index time series.

We collected 1,150,362 COVID-19–related tweets that originated from Ontario, Canada, between the period of March 12 and October 31, 2020, which consisted of 235 days. The mean daily tweet frequency was 4933 (SD 1065). The descriptive statistics and bivariate associations for outcomes and regressors are presented in Table 2. Kruskal-Wallis one-way analysis of variance tests were conducted to identify significant differences in mean tweet frequency, aggregate sentiment, and Gini index across levels of the regressors. The mean Gini index was 24.19 (SD 0.85), meaning that, on average, 24.18% of the scores would have to be redistributed for every tweet to have the same level of sentiment. The aggregate positive to negative tweet ratio was 34.57 (SD 7.92), meaning that more tweets were considered positive than negative based on the sentiment analysis. The univariate time series for frequency of tweets, the aggregate sentiment score, and the Gini index are displayed in Figure 2.

**Figure 3:**
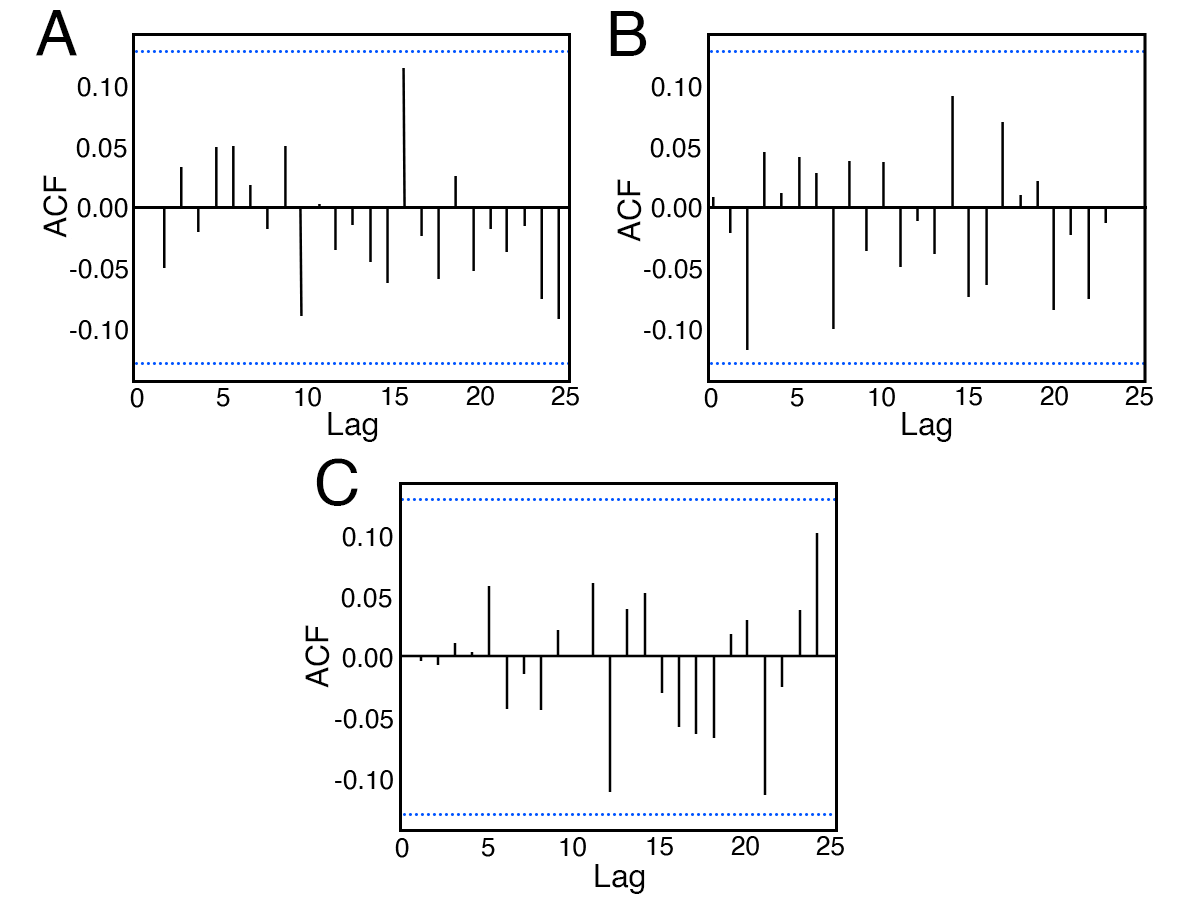
Autocorrelation function (ACF) plots for (A) COVID-19–related tweet frequency, (B) aggregate sentiment, and (C) Gini index.

After deseasonalizing the three outcome variables, augmented Dickey-Fuller and Kwiatkowski-Phillips-Schmidt-Shin tests returned large *P* values (*P*>.10) across all three variables, which provides evidence that they were stationary. This is further confirmed through visual inspection, and the fact that the auto.arima() did not require the inclusion of differencing terms in any subsequent models. An autocorrelation function plot of each outcome (Figure 3) shows no significant autocorrelations, indicating that the residuals behave like white noise and, therefore, do not exhibit temporal autocorrelations.

**Table 2:** Descriptive statistics and bivariate associations for outcomes and regressors.

Outcomes and regressors			Days with event, March 12 to October 31 (n=235), n (%)		Tweet frequency (days with condition)				Gini index (days with condition)					Positive to negative ratio (days with condition)		
					Mean (SD)		*P* value^a^		Mean (SD)		*P* value^a^		Mean (SD)			*P* value^a^
**Business closure**																
	Nonessential businesses closed	143 (60.9)		5384.90 (1136.55)		<.001		24.33 (0.78)		<.001		35.28 (10.22)			.25	
	Nonessential businesses open	92 (39.1)		4127.85 (1285.98)				23.95 (0.92)				33.46 (10.98)				
**School closure**																
	Schools open	126 (53.6)		4302.24 (1222.53)		<.001		24.03 (0.90)		.003		33.27 (10.84)			.045	
	Schools closed due to COVID-19	109 (46.4)		5575.42 (1141.91)				24.36 (0.76)				36.06 (10.03)				
**Additional restrictions**																
	No restriction announcements	223 (94.9)		4876.51 (1354.63)		.45		24.22 (0.84)		.003		34.23 (10.62)			.04	
	New or updated restriction announced	12 (5.1)		5195.16 (1122.22)				23.46 (0.75)				40.83 (6.40)				
**Regional differences in lockdown**																
	Province-wide lockdown	169 (71.9)		5137.50 (1334.11)		<.001		24.23 (0.85)		.17		34.44 (9.85)			.08	
	Partial lockdown	61 (23.0)		4347.68 (1094.77)				24.08 (0.85)				35.66 (12.07)				
	No regions under lockdown	5 (2.1)		3271.60 (1587.16)				23.76 (0.93)				25.34 (10.76)				
**Statutory holidays**																
	Holidays (with attached weekends)	17 (7.2)		4016.06 (1249.85)		.005		24.86 (0.52)		<.001		24.41 (9.34)			<.001	
	Nonholidays	218 (92.8)		4961.15 (1329.05)				24.13 (0.85)				35.36 (10.23)				
**New COVID-19 case counts (in hundreds of cases)**																
	Low (0-1.57)	78 (33.2)		4158.34 (963.00)		<.001		24.05 (0.73)		.18		35.01 (10.15)			.40	
	Medium (1.58-4.04)	76 (32.3)		5275.28 (1282.49)				24.21 (0.89)				35.51 (10.21)				
	High (4.05+)	81 (34.5)		5241.12 (1434.39)				24.28 (0.92)				33.25 (11.20)				
**New COVID-19 case counts in Canada (in hundreds of cases)**																
	Low (0-4.53)	78 (33.2)		4185.12 (1143.35)		<.001		24.22 (0.75)		.17		33.70 (10.67)			.24	
	Medium (4.54-12.36)	77 (32.8)		5174.81 (1211.07)				24.08 (0.95)				36.06 (10.23)				
	High (12.37+)	80 (34.0)		5311.31 (1382.81)				24.26 (0.86)				33.99 (10.70)				
**Official announcements of COVID-19 developments (WHO^b^ declarations, release of reopening plans, etc)**																
	No announcement	218 (92.8)		4848.34 (1342.49)		.10		24.22 (0.01)		.01		34.35 (10.31)			.21	
	Announcement	17 (7.2)		5462.65 (1258.50)				23.69 (0.92)				37.34 (13.27)				

^a^*P* values were calculated for Kruskal-Wallis tests for differences in means across levels.

^b^WHO: World Health Organization.

### COVID-19–Related Tweet Frequency

**Figure 4:**
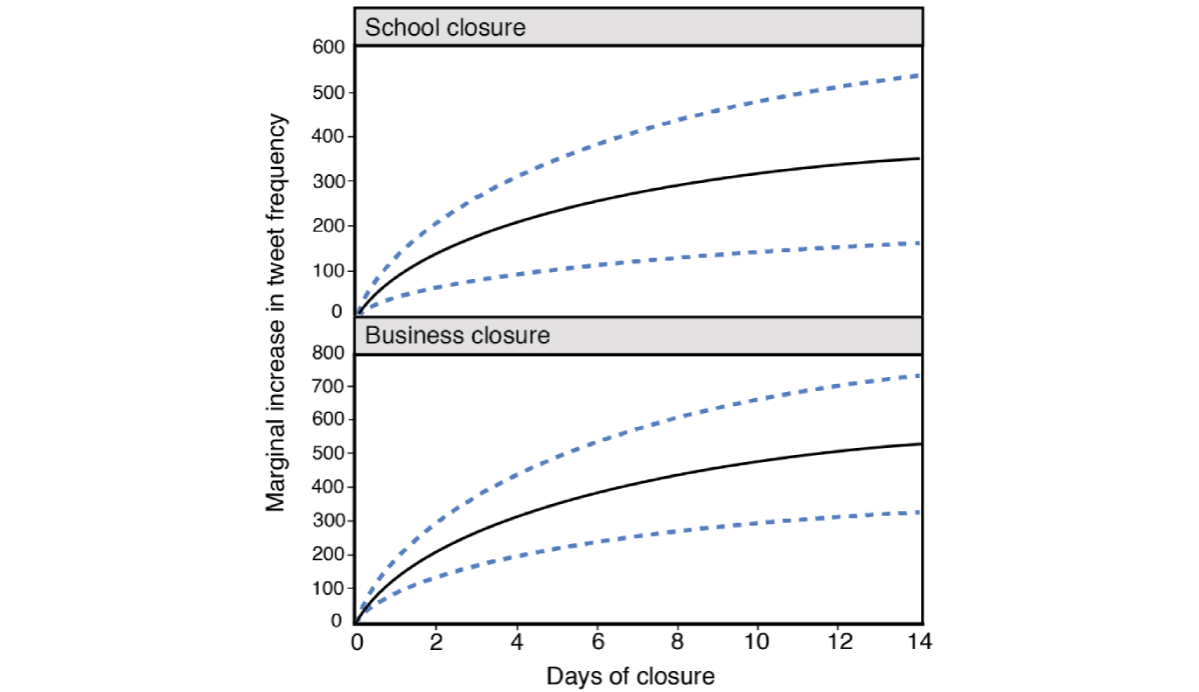
Estimated marginal increases in tweet frequency associated with increases in number of days of business and school closures, holding all other factors constant. The black line represents the estimated change in positive to negative sentiment ratio, and the dotted blue lines represent the 95% confidence interval.

Auto.arima() selected two AR terms (2, 0, 0) to be used in the ARIMA model predicting COVID-19–related tweet frequency (Table 3). Inclusion of predictor terms in the model improved the value of the AIC by 5%. Additional days of business and school closures were associated with more tweets in a nonlinear manner, where one additional day of closure had a stronger effect in the earlier part of the closure compared to the later parts. In other words, the effect of closure had a diminishing effect on tweet frequency with each additional day of closure. Each 10% increase in the duration of business closure (ie, 196 × *log_e_* [1.1] = 18.6) was associated with an increase of 18.6 tweets (95% CI 11.5-25.8). Each 10% increase in the duration of school closure (ie, 130 × *log_e_* [1.1] = 12.3) was associated with an increase of 12.3 tweets (95% CI 5.7-18.9). Figure 4 plots the rate of increase of tweet frequency associated with business and school closures. The announcement of additional public health restrictions was associated with 544 additional tweets (95% CI 178-910). Based on the statistically significant interaction between new daily COVID-19 cases in Ontario and lockdown condition (ie, Ontario case counts by province-wide vs partial lockdown; *P*<.001), new COVID-19 cases had a different effect on tweet frequency depending on the lockdown condition. Under province-wide lockdown, each increment of 100 new COVID-19 cases was associated with 391 additional tweets, and under partial lockdown, each increment of 100 new cases was associated with 134 additional tweets. The effect of new COVID-19 cases under the no lockdown condition was not different compared to the province-wide lockdown condition (*P*=.49). Compared to nonholidays, statutory holidays and their connected weekends saw a decrease of 385 tweets (95% CI –761 to –7.8). Each additional increment of 100 new cases across Canada, excluding Ontario, was associated with 46.2 additional tweets (95% CI 20.9-71.6). Days with an official COVID-19–related update saw an additional 373 tweets (95% CI 95.4-650) compared to days without any updates.

**Table 3:** Model 1: dynamic regression model predicting daily tweet frequency with ARIMA^a^ error term (2, 0, 0).

Measures				Tweet frequency	*P* value
**Predictors of daily tweet frequency, estimate of effect (95% CI)**					
	Intercept		3143 (2837 to 3450)		<.001
	Statutory holidays (1 for holidays, 0 for nonholidays)		–385 (–761 to –7.8)		.04
	Business closure (increase in 1 log day)		196 (121 to 271)		<.001
	School closure		130 (60.1 to 199)		<.001
	Additional measures		544 (178 to 910)		.003
	New COVID-19 case counts (in hundreds of cases)		391 (311 to 470)		<.001
	New COVID-19 case counts in Canada, excluding Ontario (in hundreds of cases)		46.20 (20.9 to 71.6)		<.001
	Official COVID-19–related updates		373 (95.4 to 650)		.008
	**Regional differences in lockdown**				
		Province-wide lockdown: regions are in the same stage of lockdown	Reference group		N/A^b^
		Partial lockdown: regions are in different stages of lockdown	140 (–343 to 624)		.53
		No lockdown: regions are not under lockdown	–440 (–1513 to 632)		.43
		Regions are in different stages of lockdown × new cases	–257 (–361 to –153)		<.001
		Regions are not in lockdown × new cases	1219 (–2161 to 4599)		.49
**Goodness of fit**					
	With covariates, AIC^c^		3693.89		N/A
	Without covariates, AIC		3873.20		N/A

^a^ARIMA: autoregressive integrated moving average.

^b^N/A: not applicable.

^c^AIC: Akaike information criterion.

### Positive to Negative Tweet Sentiment Ratio

**Figure 5:**
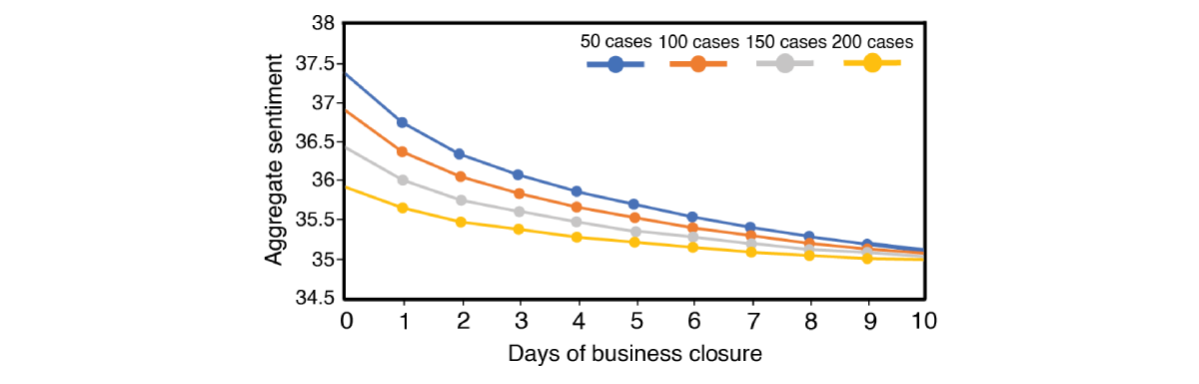
Predicted change in positive to negative sentiment ratio from day 0 to day 10 of a business closure period, varying by new COVID-19 case counts in Ontario (holding all other factors constant).

One AR term was used in the (1, 0, 0) ARIMA model predicting positive to negative tweet sentiment ratio (Table 4). Compared to the empty model, the inclusion of predictor variables improved the model AIC by 6.5%. While higher COVID-19 case counts in Ontario had the effect of reducing the positive to negative ratio of tweet sentiment, where each increment of 100 new cases was associated with –0.98 in the aggregate sentiment ratio (95% –1.81 to –0.16) during the period where no business closures were in effect, the impact of new Ontario COVID-19 cases on the sentiment ratio changed once business closures were introduced, as indicated by the significant interaction term in Table 4 (ie, business closed × Ontario new cases; *P*=.02). To facilitate interpretation of the three-way nonlinear relationship, we plotted the change in predicted aggregate sentiment ratio from day 0 to day 10 of a business closure period given four case-count scenarios, where case counts were held constant at 50, 100, 150, and 200 over the closure period, as shown in Figure 5. In short, given everything else being equal, while higher case counts reduced sentiment ratio in a direct manner, higher case counts also reduced the negative effect associated with an additional day of business closure. Compared to days when Ontario was in a province-wide lockdown, a partial lockdown was associated with an increase in sentiment ratio of 5.75. The sentiment ratio was lower on statutory holidays compared to nonholidays (–6.22, 95% CI –10.3 to –2.12). New COVID-19 cases across Canada, excluding Ontario, were not associated with a change in sentiment ratio.

**Table 4:** Model 2: dynamic regression model predicting positive to negative ratio with ARIMA^a^ error term (1, 0, 0).

Measures			Positive to negative ratio	*P* value
**Predictors of positive to negative ratio, estimate of effect (95% CI)**				
	Intercept		37.90 (34.60 to 41.20)	<.001
	Statutory holidays (1 for holidays, 0 for nonholidays)		–6.22 (–10.30 to –2.12)	.002
	Business closure (log transformed)		–1.14 (–2.26 to –0.01)	.046
	**Regional differences in lockdown**			
		Regions are in the same stage of lockdown	Reference group	N/A^b^
		Regions are in different stages of lockdown	5.75 (2.16 to 9.33)	.001
		Regions are not under lockdown	–10.50 (–18.70 to –2.29)	.01
	New COVID-19 case counts in Canada, excluding Ontario (in hundreds of cases)		0.17 (–0.14 to 0.48)	.29
	New COVID-19 case counts (in hundreds of cases)		–0.98 (–1.81 to –0.16)	.02
	Business closed × new cases (increase of 1 log unit in business closure + 100 new cases)		0.37 (0.04 to 0.70)	.02
**Goodness of fit**				
	With covariates, AIC^c^		1612.43	N/A
	Without covariates, AIC		1723.89	N/A

^a^ARIMA: autoregressive integrated moving average.

^b^N/A: not applicable.

^c^AIC: Akaike information criterion.

### Sentiment Disparity Measured by the Gini Index

One AR and two MA terms (1, 0, 2) were used in the ARIMA model predicting sentiment disparity (Table 5). Compared to the empty model (ie, with no predictors), the inclusion of predictors improved the model’s AIC value by 20%. A higher Gini index represents a more polarized range of sentiments across COVID-19–related tweets in a given day (ie, more disparities in the sentiment scores). Each 10% increase in the duration of business closure (ie, 0.113 × *log_e_* [1.1] = 0.0107) was associated with an increased Gini index of 0.01 (95% CI 0.005-0.01). Compared to days with a province-wide lockdown, days with a partial lockdown were associated with a 0.738 reduction in the Gini index (95% CI –1.19 to –0.283). We also found evidence that lockdown conditions can modify the effect of new COVID-19 case counts in Ontario on the Gini index, where each increment of 100 new cases was associated with a decrease of 0.11 in the Gini index (95% CI 0.01-0.21) while Ontario was under partial lockdown, but the Gini index remained unchanged with additional COVID-19 cases under province-wide lockdown (0.00, 95% CI –0.07 to 0.07). The Gini index was higher on statutory holidays compared to nonholidays (0.44, 95% CI 0.08-0.81). New COVID-19 cases in Canada, excluding Ontario, were not associated with the Gini index (95% CI –0.04 to 0.01).

**Table 5:** Model 3: dynamic regression model predicting the Gini index with ARIMA^a^ error term (1, 0, 2).

Measures				Gini index		*P* value
**Predictors of the Gini index, estimate of effect (95% CI)**						
	Intercept			23.90 (23.60 to 24.20)		<.001
	Statutory holidays (1 for holidays, 0 for nonholidays)			0.44 (0.08 to 0.81)		.02
	Business closure			0.11 (0.05 to 0.17)		<.001
	**Regional differences in lockdown**					
		Regions are in the same stage of lockdown	Reference group		N/A^b^	
		Regions are in different stages of lockdown	–0.738 (–1.19 to –0.28)		.001	
		Regions are not under lockdown	0.16 (–0.89 to 1.22)		.77	
	New COVID-19 case counts in Canada, excluding Ontario (in hundreds of cases)			–0.01 (–0.04 to 0.01)		.31
	New COVID-19 case counts (in hundreds of cases)			0.00 (–0.07 to 0.07)		.99
	Regions are in different stages of lockdown × new cases			0.11 (0.01 to 0.21)		.02
	Regions are not under lockdown × new cases			–1.98 (–5.06 to 1.10)		.21
**Goodness of fit**						
	With covariates, AIC^c^			461.82		N/A
	Without covariates, AIC			573.98		N/A

^a^ARIMA: autoregressive integrated moving average.

^b^N/A: not applicable.

^c^AIC: Akaike information criterion.

## Discussion

### Principal Findings

Our study found significant associations between COVID-19 restrictions and public opinion. In summary, additional days of business closures were associated with collective attention (ie, COVID-19–related tweet frequency) and increased levels of disagreement (ie, sentiment polarity). While business closures reduced aggregate sentiment (ie, the net number of tweets with positive sentiment), additional COVID-19 cases reduced the impact of business closures on overall sentiment. In other words, the model shows that people were more accepting of additional business closure days if the cases were high. While additional days of school closures were associated with collective attention, with diminishing effects for each additional day, school closures were not associated with aggregate sentiment or levels of disagreement.

Compared to province-wide lockdowns, partial lockdowns were associated with increased aggregate sentiment (ie, net number of tweets with positive sentiment) and decreased levels of disagreement. Partial lockdowns, compared to province-wide lockdowns, were associated with decreased collective attention; they also reduced the effect of additional COVID-19 case counts on collective attention. In other words, while new COVID-19 case counts increased collective attention, this effect was reduced under partial lockdown compared to province-wide lockdown. Finally, we found that the announcement of other restrictions (eg, social distancing, masking, and travel restrictions) led to increased collective attention but were not associated with changes in aggregate sentiment or level of disagreement.

### Comparison With Prior Literature

While our study was focused on investigating the unique impact of multiple pandemic restrictions on changes in public opinion over time, which has not been examined in prior literature, we found that the association between new COVID-19 case counts and collective attention—one of our ancillary findings—was consistent with prior studies, including the impact of new daily cases on Australian tweets [11] as well as the impact of daily COVID-19 incidence on Reddit posts and comments across the United Kingdom, the United States, Canada, and Italy [12].

### Limitations and Strengths

Twitter users may not be representative of the Canadian general population; therefore, our results may not be generalizable to the average Canadian. However, as of 2018, more than 15 million Canadians were classified as regular Twitter users (ie, use at least once per month) and represent a significant proportion of the 37 million members of the Canadian population [42]. One study found that North American Twitter users were younger, were more educated, and had higher income compared to the general population, but noted that their views were largely similar to the general population, except for their tendency to believe in the existence of gender and racial inequalities, which were lower in the general population [43]. In light of this information, we can interpret our findings as generalizable to a large portion of Canadians, especially for those who are younger, are more educated, have higher socioeconomic status, and tend to be more socially progressive.

Since our collection of tweets were based on keywords, there may be tweets that only contain less popular COVID-19–related keywords, such as “covidiots” or “antimask,” but do not contain common words, such as “COVID19” or “coronavirus.” While VADER has been specifically validated to analyze the sentiment in social media text, it is restricted to English-only tweets, and tweets written in other languages were not analyzed in our study. Finally, our study was not able to disentangle the separate effects of masking, social distancing, and travel restrictions, since (1) they all had similar start dates, (2) they were in effect for most of the study period, and (3) these restrictions were not lifted before the end of the study period. The overlapping nature of these restrictions limited our ability to investigate the unique contribution of their respective effects on public opinion.

Strengths of our study include the following:

1. The use of a multivariate statistical method to disentangle the effects of different pandemic restrictions; this provided stronger evidence for inference compared to prior literature studies that were largely descriptive in nature, which focused on documenting the tweet frequency and sentiment that coincided with COVID-19–related events [3,4,11].
2. Our study demonstrated the feasibility of using sentiment analysis to evaluate the impact of public health restrictions on public opinion, which can provide a relatively rapid and low-cost method to evaluate the impact of public health interventions compared to survey research.
3. We developed a novel approach of using the Gini index to measure sentiment polarization, where the index has been previously limited in its use as a measure of income disparity. Future studies may rely on the Gini index as a measure of sentiment polarization or level of disagreement.
4. Compared to prior studies that tended to focus only on the association between COVID-19–related events and collective attention, as measured by tweet frequency, our study examined the effect of restrictions on multiple dimensions of public opinion, including collective attention, aggregate sentiment, and level of disagreement, which provides a more holistic perspective of public opinion compared to single-measure studies.

### Conclusions

Our study demonstrates the feasibility of combining sentiment analysis of social media text with dynamic regression models to understand the relationship between the introduction of COVID-19 restrictions and changes in public opinion over time, which provides a rapid and flexible method of evaluating the public response to large-scale restrictions. Our study also offers useful insights on the public opinion of COVID-19 restrictions; specifically, we showed that the impact of restriction on public opinion was contextually driven (eg, business closures were better tolerated with higher COVID-19 case counts), and while school closures and other restrictions generated increased collective attention, they did not have an effect on aggregate sentiment or the level of disagreement. Partial lockdowns were associated with better public response (ie, higher number of tweets with net positive sentiment and lower levels of disagreement) compared to province-wide lockdowns. This information can help public health practitioners anticipate public response to future pandemic restrictions and ensure adequate resources are dedicated to addressing increases in negative sentiment and levels of disagreement in the face of scientifically informed, but controversial, restrictions.

## Appendix

Multimedia Appendix 1 Timeline of key COVID-19–related events in Ontario from March to October 2020.

## Abbreviations

AIC: Akaike information criterion
AR: autoregressive
ARIMA: autoregressive integrated moving average
MA: moving average
VADER: Valence Aware Dictionary and Sentiment Reasoner

## References

[1] Rufai SR, Bunce C (2020). World leaders' usage of Twitter in response to the COVID-19 pandemic: a content analysis. J Public Health (Oxf).

[2] Tsao S, Chen H, Tisseverasinghe T, Yang Y, Li L, Butt ZA (2021). What social media told us in the time of COVID-19: A scoping review. Lancet Digit Health.

[3] Valdez D, Ten Thij M, Bathina K, Rutter LA, Bollen J (2020). Social media insights into US mental health during the COVID-19 pandemic: Longitudinal analysis of Twitter data. J Med Internet Res.

[4] Chandrasekaran R, Mehta V, Valkunde T, Moustakas E (2020). Topics, trends, and sentiments of tweets about the COVID-19 pandemic: Temporal infoveillance study. J Med Internet Res.

[5] Hou K, Hou T, Cai L (2021). Public attention about COVID-19 on social media: An investigation based on data mining and text analysis. Pers Individ Dif.

[6] Zhu B, Zheng X, Liu H, Li J, Wang P (2020). Analysis of spatiotemporal characteristics of big data on social media sentiment with COVID-19 epidemic topics. Chaos Solitons Fractals.

[7] Nguyen TT, Criss S, Dwivedi P, Huang D, Keralis J, Hsu E, Phan L, Nguyen LH, Yardi I, Glymour MM, Allen AM, Chae DH, Gee GC, Nguyen QC (2020). Exploring US shifts in anti-Asian sentiment with the emergence of COVID-19. Int J Environ Res Public Health.

[8] Lwin MO, Lu J, Sheldenkar A, Schulz PJ, Shin W, Gupta R, Yang Y (2020). Global sentiments surrounding the COVID-19 pandemic on Twitter: Analysis of Twitter trends. JMIR Public Health Surveill.

[9] Kurten S, Beullens K (2021). #Coronavirus: Monitoring the Belgian Twitter discourse on the severe acute respiratory syndrome coronavirus 2 pandemic. Cyberpsychol Behav Soc Netw.

[10] Wang H, Li Y, Hutch M, Naidech A, Luo Y (2021). Using Tweets to understand how COVID-19-related health beliefs are affected in the age of social media: Twitter data analysis study. J Med Internet Res.

[11] Yigitcanlar T, Kankanamge N, Preston A, Gill PS, Rezayee M, Ostadnia M, Xia B, Ioppolo G (2020). How can social media analytics assist authorities in pandemic-related policy decisions? Insights from Australian states and territories. Health Inf Sci Syst.

[12] Gozzi N, Tizzani M, Starnini M, Ciulla F, Paolotti D, Panisson A, Perra N (2020). Collective response to media coverage of the COVID-19 pandemic on Reddit and Wikipedia: Mixed-methods analysis. J Med Internet Res.

[13] Lewandowsky S, Jetter M, Ecker UKH (2020). Using the president's tweets to understand political diversion in the age of social media. Nat Commun.

[14] Banda J, Tekumalla R, Wang G, Yu J, Liu T, Ding Y A large-scale COVID-19 Twitter chatter dataset for open scientific research -- An international collaboration. ArXiv..

[15] Tekumalla R, Banda JM (2020). Social Media Mining Toolkit (SMMT). Genomics Inform.

[16] GeoNames.

[17] Chumlab (2020). COVID-19 Project. GitHub.

[18] Hutto C, Gilbert E (2014). VADER: A parsimonious rule-based model for sentiment analysis of social media text. Proceedings of the 8th International AAAI Conference on Weblogs and Social Media.

[19] Botchway R, Jibril A, Kwarteng M, Chovancova M, Oplatková Z (2019). A review of social media posts from UniCredit bank in Europe: A sentiment analysis approach. Proceedings of the 3rd International Conference on Business and Information Management.

[20] Crocamo C, Viviani M, Famiglini L, Bartoli F, Pasi G, Carrà G (2021). Surveilling COVID-19 emotional contagion on Twitter by sentiment analysis. Eur Psychiatry.

[21] Elbagir S, Yang J (2019). Twitter sentiment analysis using Natural Language Toolkit and VADER sentiment. Proceedings of the 27th International MultiConference of Engineers and Computer Scientists.

[22] Sprenger TO, Tumasjan A, Sandner PG, Welpe IM (2013). Tweets and trades: The information content of stock microblogs. Eur Financ Manage.

[23] Mao H, Counts S, Bollen J (2015). Quantifying the Effects of Online Bullishness on International Financial Markets. European Central Bank.

[24] Simonoff J, Sparrow I (2000). Predicting movie grosses: Winners and losers, blockbusters and sleepers. Chance.

[25] Asur S, Huberman B (2010). Predicting the future with social media. Proceedings of the IEEE/WIC/ACM International Conference on Web Intelligence and Intelligent Agent Technology.

[26] Kovacevic M (2010). Measurement of Inequality in Human Development – A Review. United Nations Development Programme.

[27] Thomas V, Wang Y, Fan X (1999). Measuring Education Inequality: Gini Coefficients of Education.

[28] Hörcher D, Graham DJ (2020). The Gini index of demand imbalances in public transport. Transportation.

[29] Manek AS, Shenoy PD, Mohan MC, Venugopal KR (2017). Aspect term extraction for sentiment analysis in large movie reviews using Gini Index feature selection method and SVM classifier. World Wide Web.

[30] (2021). COVID-19 intervention timeline in Canada. Canadian Institute for Health Information.

[31] (2020). Enhanced Epidemiological Summary: COVID-19 in Ontario: A Summary of Wave 1 Transmission Patterns and Case Identification. Public Health Ontario.

[32] Nielsen K (2020). A timeline of COVID-19 in Ontario. Global News.

[33] (2020). A timeline of events in Canada's fight against COVID-19. CP24.

[34] (2020). The Daily: Canada's population estimates: Subprovincial areas, July 1, 2019. Statistics Canada.

[35] Slaughter G (2020). Canadians prefer strict lockdowns over partial shutdowns for hard-hit regions: Nanos survey. CTV News.

[36] Hu W (2013). Real-time Twitter sentiment toward Thanksgiving and Christmas holidays. Soc Netw.

[37] Jackson H (2020). Canada could see "grotesque" spike in coronavirus cases after holidays: Expert. Global News.

[38] CSSEGISandData (2021). COVID-19 Data Repository by the Center for Systems Science and Engineering (CSSE) at Johns Hopkins University. GitHub.

[39] Jebb AT, Tay L, Wang W, Huang Q (2015). Time series analysis for psychological research: Examining and forecasting change. Front Psychol.

[40] Pankratz A (2012). Forecasting with Dynamic Regression Models.

[41] Woodward W, Gray H, Elliott A (2017). Applied Time Series Analysis With R. 2nd edition.

[42] Slater M (2018). By the numbers: Twitter Canada at Dx3 2018. Twitter.

[43] Wojcik S, Hughes A (2019). Sizing Up Twitter Users. Pew Research Center.

