# Supplementary material for: Changes in Public Response Associated With Various COVID-19 Restrictions in Ontario, Canada: Observational Infoveillance Study Using Social Media Time Series Data
Source: J Med Internet Res. 2021 Aug 25;23(8):e28716. doi: 10.2196/28716 (PMC8396548; doi:10.2196/28716)
Supplement: Multimedia Appendix 1 [file jmir_v23i8e28716_app1.docx]

Multimedia Appendix 1

Table S1: Timeline of key COVID-related events in Ontario from March to October 2020

|  | COVID-related events and restrictions |
| --- | --- |
|  |  |
| **Date (m/d)** |  |
| 3/11 | Announcement from WHO regarding COVID-19 outbreak as a pandemic |
| 3/12 | Announcement that Ontario schools are to be closed |
| 3/14 | Federal Government strongly urges abroad canadians to return home |
| 3/17 | Ontario's first death attributed to COVID-19  State of emergency announced in Ontario; the following establishments are legally required to close immediately:  All facilities providing indoor recreational programs; All public libraries; All private schools as defined in the Education Act; All licensed child care centres; All bars and restaurants, except to the extent that such facilities provide takeout food and delivery; All theatres including those offering live performances of music, dance, and other art forms, as well as cinemas that show movies; and Concert venues. |
| 3/18 | Announcement that US Canada non-essential travel restricted |
| 3/21 | US Canada border closed |
| 3/23 | Announcement that Non-essential businesses are ordered closed for 14 days starting midnight next day |
| 3/25 | Announcement of CERB |
| 3/27 | Ford government issues an emergency alert to peoples phones warning recent travellers to self-isolate |
| 3/28 | Announcement and implementation of no more than 5 people at gatherings |
| 3/30 | Province orders shutdown of all outdoor amenities |
| 3/31 | Ontario schools will remain closed until at least May |
| 4/3 | Province releases first projections, suggesting 1600 deaths and 80k cases by end of April  List of non-essential businesses extended |
| 4/6 | CERB sign up begins |
| 4/8 | Ontario orders grocery stores and pharmacies to be closed on friday and sunday of the holiday weekend |
| 4/14 | Ontario state of emergency extended for another four weeks |
| 4/22 | Ontario government requests military aid for COVID-struck long-term care homes |
| 4/25 | Ontario Announces Pandemic Pay for Frontline Workers (Eligible workers will receive a $4 per hour premium on top of their regular hourly wages) |
| 4/26 | Publically funded schools will be closed until at least May 31 |
| 4/27 | Doug Ford provides official framework in which he will reopen the province with no starting date |
| 5/1 | Ontario government announces that some seasonal businesses (including nurseries) and some essential construction projects can open on May 4 if they follow strict guidelines. |
| 5/6 | Emergency orders are extended for third time until May 19 |
| 5/9 | Province announces that beginning on May 11, residents are allowed to walk, hike, bike and bird watch in provincal parks. Camping and access to beaches will remain closed. |
| 5/14 | Doug Ford provides details on province's first stage of its Phase 2: Restart (in effect may 16 & 19) |
| 5/16 | First stage of Phase 2: Restart part I |
| 5/19 | All regions enter stage 1 of reopening plan Ontario government says in-class school year not happening |
| 5/26 | Documents released about the poor conditions in LTC homes |
| 5/27 | Ontario extends emergency orders until June 9th Province announces that chiropractors, dentists, massage therapists, optometrists, homeopaths and psychologists can see patients once each profession gets the go-ahead and receives guidelines from each respective governing authority. |
| 6/2 | Ontario extends emergency orders for another 28 days |
| 6/5 | George Floyd protests in Canada (Ottawa Parliament hill about 20k ppl) |
| 6/8 | Ontario announces Stage 2 of recovery plan (Toronto, Mississauga and Hamilton are among the areas which are excluded) |
| 6/9 | Province announces daycares can open the following Friday although few do |
| 6/12 | Social gathering limits increase from 5 to 10, except for Toronto |
| 6/14 | Ontario announces it has conduced more than 1m tests |
| 6/15 | Province announces that several regions including Durham, York, Hamilton, Sarnia-Lambton and Niagara can enter stage 2 of recovery plan |
| 6/17 | Ontario extends emergency orders until June 30 |
| 6/18 | Visitors allowed back into LTC homes Canada officially records more than 100,000 cases of COVID-19 over the length of the pandemic. |
| 6/22 | Toronto and Peel permitted to enter stage 2. Windsor-Essex remains alone in stage 1 |
| 6/24 | Ontario extends emergency orders until July 15, most of Windsor-Essex move to stage 2, Leamington and Kingsville left in stage 1 |
| 6/28 | summer break starts |
| 7/1 | Province asks school boards to consider starting the school year early |
| 7/6 | Kingsville and Leamington move to stage 2, whole province now in stage 2 |
| 7/9 | Ontario extends emergency orders until July 22 |
| 7/13 | Ontario announces some of province will enter stage 3 on July 17. Toronto left in stage 2 |
| 7/16 | Ontario to extend state of emergency to July 29 |
| 7/17 | 24 regions move into stage 3 |
| 7/24 | Hamilton, York, Durham, Halton, Niagara and others move to stage 3 |
| 7/28 | Remdesivir officially becomes the first drug to be approved by Health Canada for treatment of patients with severe COVID-19 symptoms. |
| 7/29 | Toronto and Peel can move to stage 3 on July 31 |
| 7/30 | Ontario announces format of online schooling |
| 7/31 | Toronto and Peel move to stage 3 |
| 8/10 | Windsor-Essex can move to stage 3 on August 12 |
| 8/14 | Ontario government announces it is increasing number of people at indoor sports, fitness and recreation facilities |
| 8/26 | Ontario government release official plan on how to deal with outbreaks in school |
| 9/4 | summer break ends |
| 9/8 | An official update from Health Minister Christine Elliott says there will be a pause in loosening any more restrictions |
| 9/17 | Ford reduces # of people in private gatherings in Toronto, Peel, and Ottawa |
| 9/18 | An official update from Public Safety Minister Bill Blair says Canada will extend the partial closure of the border with the U.S. for another month. |
| 9/19 | Private gatherings reduced across province |
| 9/20 | Official update from the Health minister says province has conducted 40k+ tests in 24h |
| 9/23 | Trudeau says the second wave of COVID-19 is already underway. He says families won't likely be able to gather for Thanksgiving, but it is not too late to save Christmas.  Ontario government announces pharmacies in select locations will begin offering COVD-19 tests to people who aren't experiencing symptoms of the virus as part of its fall prepardness plan |
| 9/25 | The province orders strip clubs to close while also ordering restaurants, bars, and other food and drink businesses to stop serving alcohol at 11 p.m |
| 9/28 | An official update from Ontario Hospital Association suggests a return to stage 2 Ontario reports highest case count since april  Ontario is officially in second wave of pandemic |
| 9/29 | Canada signs deal to buy 8m rapid tests |
| 9/30 | Parliamentarians unanimously pass Bill C-4 to usher in a new batch of COVID-19 benefits. For Canadians left jobless or underemployed because of the pandemic, the legislation supplants the CERB support program with a more flexible and generous employment insurance regime. |
| 10/1 | Province announces new screening guidelines for daycares and schools |
| 10/2 | Ontario announces province-wide mask policy and announces new restrictions on restaurants, bars, banquet halls, and gyms in ottawa, Toronto, Peel (in effect oct 3) |
| 10/5 | Doug Ford rejects calls for indoor dining to be ordered closed province switches to by appointment testing only - most test centres are full |
| 10/9 | New restrictions are put in place for Toronto, Peel Region and Ottawa amid rising coronavirus cases including the closure of indoor dining at restaurants and bars, as well as the closure of gyms, casinos, cinemas, and performing arts centres. |
| 10/16 | Ford announced York Region roll back to Modified Stage 2 for 28 days from Oct 19 |
| 10/19 | Ontario recommends no Trick or Treating  Canada's COVID-19 case count surpasses the 200,000 mark. The development comes just over four months after Canada reached the 100,000-case threshold. |
| 10/23 | Official update from Ontario’s independent commission into long-term care releases early recommendations for the Ford government to implement during the second wave of the pandemic |
| 10/25 | Ontario officially reports over 1,000 daily new cases of COVID-19 for the first time |

^a^Sourced from: Canadian Institute for Health Information^21^, Public Health Ontario^22^, Global News^23^, and CP24^23^.
